# Frozen tissue coring and layered histological analysis improves cell type-specific proteogenomic characterization of pancreatic adenocarcinoma

**DOI:** 10.1186/s12014-024-09450-3

**Published:** 2024-01-30

**Authors:** Sara R. Savage, Yuefan Wang, Lijun Chen, Scott Jewell, Chelsea Newton, Yongchao Dou, Qing Kay Li, Oliver F. Bathe, Ana I. Robles, Gilbert S. Omenn, Mathangi Thiagarajan, Hui Zhang, Galen Hostetter, Bing Zhang

**Affiliations:** 1 Lester and Sue Smith Breast Center, Baylor College of Medicine, Houston, TX 77030 USA; 2 Department of Molecular and Human Genetics, Baylor College of Medicine, Houston, TX 77030 USA; 3 Department of Pathology, Johns Hopkins University, Baltimore, MD 21231 USA; 4 Van Andel Institute, Grand Rapids, MI 49503 USA; 5 Departments of Surgery and Oncology, Cumming School of Medicine, University of Calgary, Calgary, AB Canada; 6 Arnie Charbonneau Cancer Institute, Calgary, AB Canada; 7 Office of Cancer Clinical Proteomics Research, National Cancer Institute, Rockville, MD 20850 USA; 8 Department of Computational Medicine & Bioinformatics, Internal Medicine, Human Genetics, and School of Public Health, University of Michigan, Ann Arbor, MI 48109 USA; 9 Leidos Biomedical Research Inc., Frederick National Laboratory for Cancer Research, Frederick, MD 21702 USA

**Keywords:** Proteogenomic, Microenvironment, Tissue coring, CPTAC

## Abstract

**Background:**

Omics characterization of pancreatic adenocarcinoma tissue is complicated by the highly heterogeneous and mixed populations of cells. We evaluate the feasibility and potential benefit of using a coring method to enrich specific regions from bulk tissue and then perform proteogenomic analyses.

**Methods:**

We used the Biopsy Trifecta Extraction (BioTExt) technique to isolate cores of epithelial-enriched and stroma-enriched tissue from pancreatic tumor and adjacent tissue blocks. Histology was assessed at multiple depths throughout each core. DNA sequencing, RNA sequencing, and proteomics were performed on the cored and bulk tissue samples. Supervised and unsupervised analyses were performed based on integrated molecular and histology data.

**Results:**

Tissue cores had mixed cell composition at varying depths throughout. Average cell type percentages assessed by histology throughout the core were better associated with *KRAS* variant allele frequencies than standard histology assessment of the cut surface. Clustering based on serial histology data separated the cores into three groups with enrichment of neoplastic epithelium, stroma, and acinar cells, respectively. Using this classification, tumor overexpressed proteins identified in bulk tissue analysis were assigned into epithelial- or stroma-specific categories, which revealed novel epithelial-specific tumor overexpressed proteins.

**Conclusions:**

Our study demonstrates the feasibility of multi-omics data generation from tissue cores, the necessity of interval H&E stains in serial histology sections, and the utility of coring to improve analysis over bulk tissue data.

**Supplementary Information:**

The online version contains supplementary material available at 10.1186/s12014-024-09450-3.

## Background

Pancreatic adenocarcinoma (PDAC) is the 3rd leading cause of cancer-related death in the United States and has a 5-year relative survival rate of only 11% [1]. PDAC is often detected at a late stage and rarely responds to conventional cancer therapies [2, 3]. One barrier to the identification of biomarkers of early tumors and the development of effective treatments for PDAC is the low tumor content and high degree of cellular heterogeneity in bulk tumor samples [4].

Transcriptomic and other molecular studies typically use bulk surgically resected PDAC tissue samples to reveal somatic mutations and dysregulated gene expression [5–7]. However, both non-neoplastic ductal epithelium and tumor epithelium comprise only a small fraction of the tumor mass. Indeed, the percent neoplastic cell content in bulk tissue samples has a median of 18–25% in several studies, suggesting a low overall signal from neoplastic epithelium in the molecular data [5, 6]. Bulk tissue analysis may miss genes important to pancreatic adenocarcinoma pathology and the differences due to variations in cell types across samples from the bulk tissues may be amplified. In silico deconvolution algorithms such as xCell [8], DECODER [9], EDec [10], and DeMixT [11] can dissect bulk omics data into cell type compartments. However, these algorithms may not perform well unless all types of cells contained in the tissue are considered, and they are often not validated with dissociated solid tissues such as the type found in omics studies. Single cell RNA sequencing can also be used to identify and evaluate different tissue types, but this technology is still under development for proteomics.

Despite the challenges in dissecting specific cellular components in PDAC tissue, analyzing separate tissue compartments is still of interest. While genes and proteins expressed by neoplastic cells provide valuable insights into tumor characteristics, the microenvironment plays an important role in supporting drug delivery, immunotherapy, and tumor growth [12, 13]. Understanding the molecular components of the stroma and epithelial cells separately could help decode the data from bulk tissue and point to targetable features for therapy. Enrichment of epithelial and stroma components from tissue can be done using physical methods such as laser capture microdissection or scrape macrodissection from tissue sections. Maurer et al. used laser capture microdissection to separately extract PDAC epithelium and stroma and performed RNAseq [14]. This method can provide mostly pure tissue compartments but is time-consuming and expensive and the laser may also introduce artifacts by heating the tissues. More recently, microscaled tissue coring was used to select specific tissue regions. This method is less time intensive and yields larger tissue amounts. The Biopsy Trifecta Extraction (BioTExt) coring method was shown to provide enough tissue for multiple analyte extractions for downstream multi-omics study with comparable results to bulk tissue [15]. We wanted to assess the feasibility of using this coring technique to generate multi-omics data for PDAC samples and identify epithelial-specific and stroma-specific differences between tumor and adjacent non-tumor samples. Because core selection is based on histology from the top region of the tissue block, a key question is whether surface histology is consistent with the rest of the cored tissue, as changes in cellular composition may lead to misinterpretation of omics data generated from the whole depth. Here, we performed BioTExt coring on tumor and adjacent non-tumor tissue blocks from 15 patients with PDAC. We performed histology at multiple layers of the tissue core and generated multi-omics data for bulk tissue, epithelial-enriched cores, and stroma-enriched cores from both tumor and adjacent samples. Our analysis of these data demonstrate that BioTExt coring in combination with layered histology facilitates cell type-aware proteogenomic characterization of PDAC.

## Methods

### Specimens and clinical data

Tumor, adjacent non-tumor tissue, and whole blood from 15 PDAC patients were used in this study. All samples were prospectively collected between April 2017 to February 2018 for the Clinical Proteomics Tumor Analysis Consortium (CPTAC) program. Biospecimens were collected from newly diagnosed patients scheduled for surgical treatment of a pancreatic mass suspected to be PDAC and had received no prior treatment for their disease, including chemotherapy or radiotherapy, and were collected independent of grade or stage. The samples were moderately differentiated and ranged between 20 to 60% tumor nuclei by standard QC tissue evaluation by H&E staining and study pathologist. Patient outcome data was collected for all cases.

### Sample processing

The CPTAC Biospecimen Core Resource (BCR) at Van Andel Research Institute (Grand Rapids, Michigan) manufactured and distributed biospecimen collection kits to the individual Tissue Source Sites (TSS). Each collection kit contained a set of pre-labeled barcodes for tracking of individual specimens respective to TSS collection site, original pathological report, and sample type. This standard procedure assured the accuracy of all samples through individual TSS to the BCR and to the CPTAC proteomic and genomic characterization centers.

Fresh tissue specimens were collected and sectioned on the longest axis into two pieces. The larger pieces, averaging a weight of 316 mg, were snap-frozen at the TSS within a 30 min cold ischemic time (CIT, average time = 19 min) and the corresponding mirror-image sections were formalin-fixed paraffin-embedded (FFPE) and H&E stained based on CPTAC guidelines. Acceptable samples were transported using a -140 °C cryoport with a time and temperature tracker to the BCR. At the BCR, examination of the specimen integrity included a physical inspection, review of the time and temperature tracker, and barcode entry into the biospecimen tracking database. All pathologic characteristics of collected tumor samples, including viable tumor nuclei (> 20%), total cellularity (> 50%), and tumor necrosis (< 20%), were re-evaluated and verified by BCR pathologists. Additionally, CPTAC disease-specific working group and pathology experts reviewed the morphology of tumor samples to confirm the diagnosis prior to proteomic and genomic analyses. DNA sequencing was performed at the Broad Institute, Cambridge, MA and RNA sequencing was performed at the University of North Carolina, Chapel Hill, NC. Material for proteomic analyses was sent to the Proteomic Characterization Center (PCC) at Johns Hopkins University, Baltimore, MD. See Additional file 1: Table S1 for sample IDs.

### BioTExt coring

Tumor blocks of treatment naïve PDAC were selected from CPTAC patient samples based on initial QC assessment on the whole section H&E. Surface cryosections were prepared from the frozen tumor blocks post embedding in optimal cutting temperature compound (OCT) and H&E stained for QC verification. Frozen tissue coring was performed using the CryoXtract method (CXT 350, Basque Engineering + Science Inc. West Newbury, MA) with rigid gauge 1.5 mm diameter needles to sample specified regions selected by the study pathologist (GH) from the H&E surface cryosection. For this study, histomorphology of neoplastic epithelium (glandular), stroma (non-acinar), and non-tumor (acinar) or mixed were recorded. The frozen bulk tissue was embedded in OCT prior to CryoCore processing to ensure intact frozen cores of 1.5 mm diameter and 3–7 mm depth. Two to four cores were extracted from bulk tumor tissue. Regions to target neoplastic glandular tissue and stroma rich (non-acinar) were selected to minimize acinar contamination and allow direct comparison of neoplastic glands and stroma rich regions.

As previously described as the BioTExt method [15], the frozen tissue core was transposed 90 degrees and embedded lengthwise in a fresh OCT mold for sequential sectioning through the 1.5 mm aspect to provide cryosections for genomic, transcriptomic, and proteomic applications. Intervening H&E sections were performed at various levels to document retention or loss of tumor or stroma or acinar contaminant in the third dimension (core depth). Depending on extracted integrity 3 to 4 H&E sections of lengthwise core were used to estimate percent tumor and stroma at each level. All cell counts were performed by the study pathologist, GH.

### Sample processing for genomic DNA and total RNA extraction

Bulk and cored samples were processed for DNA and RNA sequencing as previously described [5]. Briefly, DNA and RNA were isolated using QIAsymphony DNA Mini Kit and RNA kit (QIAGEN) according to instructions from the manufacturer.

### DNA library construction

Library construction was performed as previously described [5]. Kapa HyperPrep reagents were used for end repair/A-tailing, adapter ligation [palindromic forked adapters (IDT)], and library enrichment PCR. Library pools were quantified using qPCR (KAPA Biosystems) and were normalized to 2 nM.

### Whole exome sequencing

Sequencing was performed as described in [5]. Flowcells were sequenced utilizing sequencing-by-synthesis chemistry and were analyzed using RTA v2.7.3 or later. Library pools were sequenced on paired 76 cycle runs and then run on HiSeq 4000-paired end runs. Data were processed and somatic mutations were called by the pipeline at the genomics data commons (GDC).

### Somatic mutation summary

We downloaded somatic mutation calls from the GDC (https://gdc.cancer.gov/, Additional file 2: Table S2). Variant allele frequency of a somatic mutation was calculated as (# of mutated reads) / (# of WT + # of mutated reads).

### RNA quality control and library construction

RNA samples were quantified on a TapeStation system (Agilent, Inc. Santa Clara, CA) and samples with RIN > 8.0 were considered high quality. Total RNAseq library construction was performed using the TruSeq Stranded RNA Sample Preparation Kit (Illumina) on an Agilent Bravo Automated Liquid Handling System. Libraries were quantified on a TapeStation system.

### Total RNA sequencing

Libraries were run on a HiSeq 4000 paired end 75 base pairs to generate at least 120 million reads per sample. Data were demultiplexed and converted to FASTQ files. Reads were mapped to the hg38 human reference genome.

### RNA quantification

The hg38 reference genome and RefSeq annotations were used for the RNAseq data analysis and were downloaded from the UCSC table browser. First, CIRI (v2.0.6) [16] was used to call circular RNA (circRNA) with default parameters and BWA (version 0.7.17-r1188) was used as the mapping tool. To remove circRNA abundance from linear transcript quantification, we used a cutoff of 10 supporting reads for circRNAs. Then we used a pseudo-linear transcript strategy to quantify gene and circular RNA expression [17]. In brief, for each sample, linear transcripts of circular RNAs were extracted and 75 bp (read length) from the 3′ end was copied to the 5′ end. The modified transcripts were called pseudo-linear transcripts. Transcripts of linear genes were also extracted and mixed with pseudo-linear transcripts. RSEM (version 1.3.1) with Bowtie2 (version 2.3.3) as the mapping tool was used to quantify gene and circular RNA expression based on the mixed transcripts. After quantification, the upper quantile method was applied for normalization. The normalized matrix was log2-transformed and separated into gene and circular RNA expression matrices.

### Sample processing for protein extraction and tryptic digestion

The PDAC BioTExt samples were processed for mass spectrometric (MS) analysis at Johns Hopkins University. Before protein extraction and digestion, tissues were washed using a stepwise ethanol gradient to eliminate the OCT. Tissues were placed in a 2-ml micro-tube and washed with 1.5 ml chilled 70% ethanol in water for 15 s with vortexing. The supernatant was then removed by centrifugation at 20,000×*g* for 5 min at 4 °C. Next, tissues were washed with 1.5 ml chilled HPLC-grade water for 15 s with vortexing followed by centrifugation at 20,000×*g* for 5 min. Then, 1.5 ml chilled 100% ethanol was added to the tissue samples followed by vortexing for 15 s and the supernatant was removed by centrifuging at 20,000×g for 5 min. Following OCT removal, tissue lysis and downstream sample preparation for global proteomic analysis were carried out as previously described [18]. Tumors or adjacent tissue BioTExt samples were homogenized separately in 100 μL lysis buffer (8 M urea, 75 mM NaCl, 50 mM Tris, pH 8.0, 1 mM EDTA, 2 μg/mL aprotinin, 10 μg/mL leupeptin, 1 mM PMSF, 10 mM NaF, Phosphatase Inhibitor Cocktail 2 and Phosphatase Inhibitor Cocktail 3 [1:100 dilution], and 20 μM PUGNAc) by repeated vortexing. Proteins in the lysates were clarified by centrifugation at 20,000×*g* for 10 min at 4 °C, and protein concentrations were determined by BCA assay (Pierce). The protein lysates were then diluted to a final concentration of 2 mg/mL in lysis buffer for reduction, alkylation, and digestion. Protein lysates (160 μg) were reduced with 5 mM dithiothreitol (DTT) for 1 h at 37 °C and subsequently alkylated with 10 mM iodoacetamide for 45 min at RT (room temperature) in the dark. Samples were then diluted by 1:4 with 50 mM Tris–HCl (pH 8.0) and subjected to proteolytic digestion with LysC (Wako Chemicals) for 2 h incubation at RT, followed by the addition of sequencing-grade modified trypsin (Promega) for overnight incubation at RT. Digested samples were then acidified with 50% formic acid (FA, Fisher Chemicals) to pH < 3. Tryptic peptides were desalted on reversed-phase C18 SPE columns (Waters) and dried using a Speed-Vac (Thermo Scientific). Desalted peptides were reconstituted in 3% ACN/0.1% FA for data-independent acquisition mass spectrometry (DIA) proteomic analysis.

### DIA proteomic data acquisition

Samples containing 50 ng peptides were separated by the EASY-nLCTM 1200 instrument of nano-flow UHPLC (Thermo Fisher Scientific™). Peptides were analyzed by reversed phase PicoFrit^®^ LC–MS Columns (New Objective, MA, USA), which were packed with 0.9 μm/120 Å ReproSil-Pur C18 resin (Dr. Maisch, Ammerbuch, Germany) at 28 cm long. Global peptides were separated over a running time of 30 min using a 6–30% gradient of buffer B (90% acetonitrile and 0.1% formic acid) at a flow rate of 200 nL/min. The MS parameters were as follows: MS1, AGC target 1 × 10^6^, resolution 120,000, maximum injection time 60 ms, isolation window 12.0 m/z, window overlap 2.0 m/z, scan range 400 − 1000 m/z; MS2, AGC target 1 × 10^6^, resolution 15,000, maximum injection time 50 ms, and 31% collision energy.

### Protein data quantification

Raw DIA data from PDAC BioTExt tissues were analyzed by the directDIA approach [19], which was a library-free method embedded in Spectronaut (version 14) with a precursor and protein q-value cutoff at 1%. The intensity of global proteins was normalized by cross-normalization function with imputation in the setting of quantification. DIA proteomics data were log2 transformed and median centered. Proteomics data are accessible from the Proteomics Data Commons (https://pdc.cancer.gov/, PDC000504).

### Principal components analysis (PCA)

Principal components analysis was performed using the *stats* package in R on scaled data.

### Differential expression

Differential expression was performed using the unpaired Student’s* t*-test. P values were adjusted using the Benjamini–Hochberg method and genes were considered significant with adjusted p < 0.01. Fold changes were calculated as the differences between the mean log2 values for each group.

### Over-representation analysis

Genes either increased or decreased in tumor compared to tumor-adjacent tissue (AT) were provided to WebGestaltR [20] using the “ORA” method and the Gene Ontology Biological Process (without redundancy) gene sets. The reference set contained all proteins identified by DIA proteomics.

### Histology-based clustering

Cell-type percentages were averaged across all histology slides and were clustered using k-means clustering with k = 3. Cell percentage differences and protein abundance differences were evaluated using one-way ANOVA with Tukey’s post-hoc test.

## Results

### Data generation

**Fig. 1:**
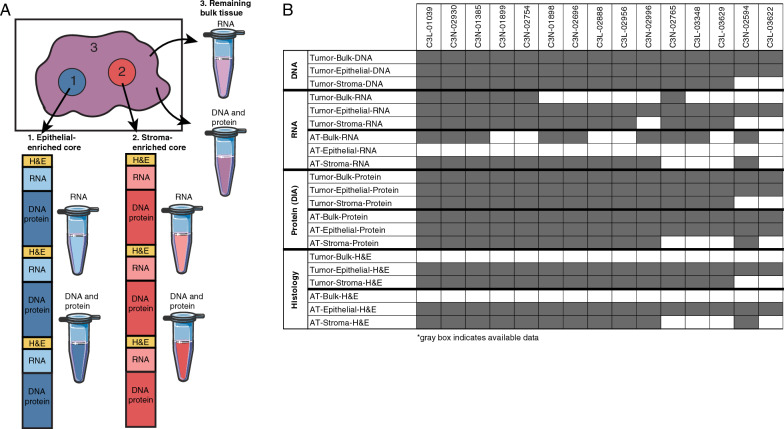
BioTExt protocol, data generation, and data availability. **A** Schematic of the BioTExt protocol. Using an H&E image from the surface of tumor tissue and adjacent tissue blocks, epithelial-enriched and stroma-enriched regions were selected for coring. The cores and the remaining bulk tissue were used for RNA, DNA, and proteomics analyses. **B** Available genomics, transcriptomics, proteomics, and histology data for tumor samples and AT samples

We collected PDAC and adjacent non-tumor tissue (AT) samples from 15 patients as part of the CPTAC program. Patient and tumor characteristics are listed in Table 1. Using an H&E-stained section from the surface of each tissue block, we selected regions enriched in epithelium or stroma tissue for coring (Fig. 1A). From the tumor blocks of each patient, three cores were collected from tumor-associated stroma tissue and three cores from neoplastic epithelial tissue. From the AT blocks, three cores were collected from the stroma tissue and three from the epithelial tissue. The cores were embedded in OCT and were serially sectioned with intervening sections for H&E staining. The compartments of all three cores not used for staining were combined and used for DNAseq, RNAseq, and DIA proteomics. The remaining bulk tissue after coring was cryopulverized and used to generate the same data types. Data availability is summarized in (Fig. 1B). Each patient had up to 6 different tissue samples for each data type. All epithelial-enriched AT cores had limited tissue and were used only for DNAseq and proteomics data generation. Other missing samples failed quality control.

**Table 1:** Patient clinical characteristics

Case ID	Age	Country of origin	Ethnicity, Self identify	Gender	BMI	Alcohol consumption	Tobacco smoking history	Tumor site	Tumor size (cm)	Histologic grade	Lymph-vascular invasion	AJCC cancer staging edition	Path stage primary tumor(pT)	Path Stage Reg Lymph nodes(pN)	Tumor stage (pathological)
C3L-01039	59	Russia	White (Caucasian)	Male	28.91	Lifelong non-drinker	Lifelong non-smoker: Less than 100 cigarettes smoked in lifetime	Head	2	G3 Poorly differentiated	Indeterminate	Seventh Edition (2010)	pT2	pN1	Stage IIB
C3L-02888	62	Canada	Norwegian	Female	26.19	Consumed alcohol in the past, but currently a non-drinker	Current smoker: Includes daily and non-daily smokers	Head	1.7	G2 Moderately differentiated	Not identified	Eighth Edition (2017)	pT3	pN0	Stage IIA
C3L-02956	84	United States	White	Female	28.83	Lifelong non-drinker	Lifelong non-smoker: Less than 100 cigarettes smoked in lifetime	Body	4.5	G2 Moderately differentiated	Present	Seventh Edition (2010)	pT3	pN1	Stage IIB
C3L-03348	37	Canada	Canadian	Male	41.8	Alcohol consumption equal to or less than 2 drinks per day for men and 1 drink or less per day for women	Lifelong non-smoker: Less than 100 cigarettes smoked in lifetime	Head	4.5	G2 Moderately differentiated	Present	Eighth Edition (2017)	pT3	pN1	Stage IIB
C3L-03622	72	Canada	Caucasian	Female	18.6	Lifelong non-drinker	Current reformed smoker within past 15 years	Head	3.5	G3 Poorly differentiated	Present	Seventh Edition (2010)	pT3	pN1	Stage IIB
C3L-03629	75	Canada	Canadian	Male	25.03	Alcohol consumption history not available	Current reformed smoker, more than 15 years	Head	2.1	G2 Moderately differentiated	Present	Seventh Edition (2010)	pT3	pN1	Stage IIB
C3N-01385	66	Poland	white	Male	28	Consumed alcohol in the past, but currently a non-drinker	Current reformed smoker, more than 15 years	Head	6	G2 Moderately differentiated	Present	Seventh Edition (2010)	pT3	pN1	Stage IV
C3N-01898	49	Poland	white	Female	25	Alcohol consumption equal to or less than 2 drinks per day for men and 1 drink or less per day for women	Lifelong non-smoker: Less than 100 cigarettes smoked in lifetime	Head	4.5	G3 Poorly differentiated	Present	Seventh Edition (2010)	pT3	pN0	Stage IIA
C3N-01899	63	Poland	white	Female	35	Lifelong non-drinker	Lifelong non-smoker: Less than 100 cigarettes smoked in lifetime	Head	3	G2 Moderately differentiated	Present	Seventh Edition (2010)	pT3	pN1	Stage IIB
C3N-02594	65	Poland	white	Female	22	Lifelong non-drinker	Lifelong non-smoker: Less than 100 cigarettes smoked in lifetime	Head	3	G2 Moderately differentiated	Present	Eighth Edition (2017)	pT2	pN0	Stage IB
C3N-02696	65	Poland	Caucasian	Male	27	Alcohol consumption equal to or less than 2 drinks per day for men and 1 drink or less per day for women	Lifelong non-smoker: Less than 100 cigarettes smoked in lifetime	Head	3	G2 Moderately differentiated	Present	Seventh Edition (2010)	pT3	pN1	Stage IIB
C3N-02754	61	China	Han	Male	22.95	Lifelong non-drinker	Lifelong non-smoker: Less than 100 cigarettes smoked in lifetime	Tail	3.5	G1 Well differentiated	Not identified	Eighth Edition (2017)	pT2	pN2	Stage III
C3N-02765	76	China	Han	Male	17.3	Lifelong non-drinker	Lifelong non-smoker: Less than 100 cigarettes smoked in lifetime	Head	4.5	G2 Moderately differentiated	Not identified	Eighth Edition (2017)	pT3	pN0	Stage IIA
C3N-02930	60	China	Han	Male	23.62	Lifelong non-drinker	Lifelong non-smoker: Less than 100 cigarettes smoked in lifetime	Head	2	G2 Moderately differentiated	Not identified	Eighth Edition (2017)	pT1c	pN2	Stage III
C3N-02996	77	Poland	Caucasian	Female	20	Lifelong non-drinker	Lifelong non-smoker: Less than 100 cigarettes smoked in lifetime	Head	4.8	G2 Moderately differentiated	Indeterminate	Eighth Edition (2017)	pT3	pN1	Stage III

### Differences between bulk, epithelial-enriched cored, and stroma-enriched cored samples

**Fig. 2:**
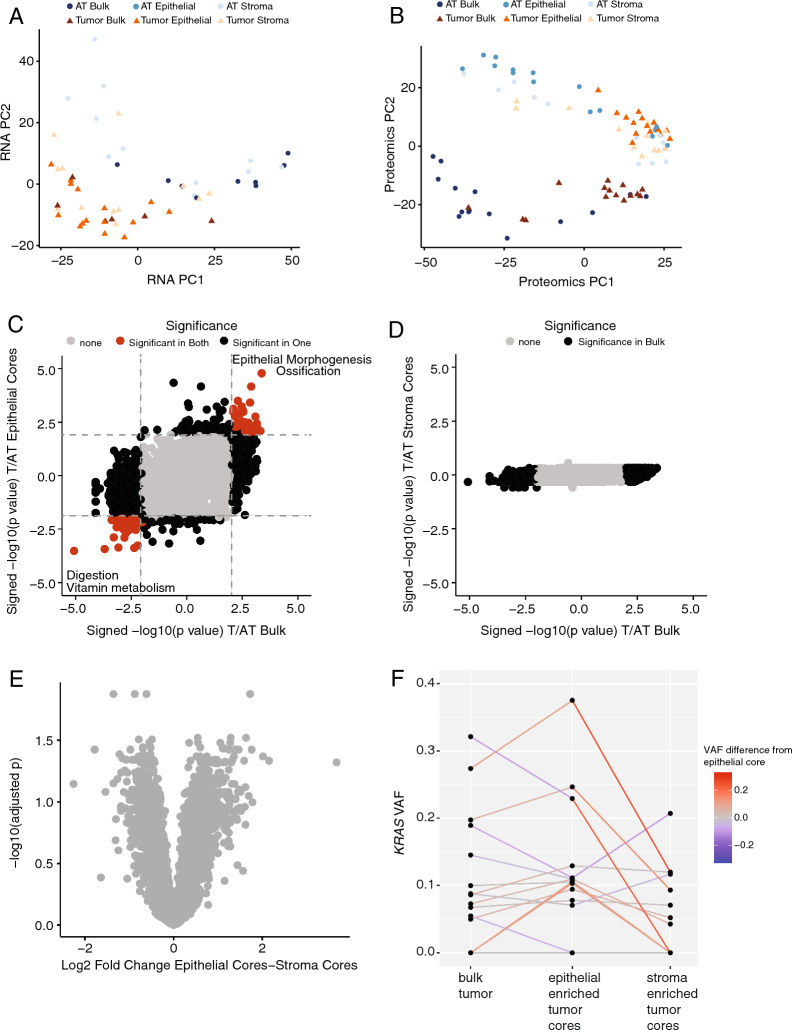
Coring enriched epithelial tissue but did not significantly differentiate tumor epithelial tissue from stromal tissue. **A** Principal component analysis for RNAseq data. All AT Epithelial samples had limited tissue availability and were not sequenced for RNA. **B** Principal component analysis for proteomics data. **C** Protein difference between tumor and AT in bulk tissue and epithelial-enriched cores. Some significantly enriched GO terms are highlighted. **D** Protein difference between tumor and AT in bulk tissue and stroma-enriched cores. **E** Difference in proteins between tumor epithelial-enriched cores and tumor stroma-enriched cores. **F** Percent increase of *KRAS* VAF from bulk tissue to epithelial-enriched cores and percent increase of *KRAS* VAF from stroma-enriched cores to epithelial-enriched cores

We quantified the expression of 34,950 genes using RNAseq and 4,903 proteins using DIA proteomics (Additional file 2: Table S2). Samples from the tumor blocks were largely separated from AT samples in the first two principal components for RNA and proteomics (Fig. 2A, B). Additionally, proteomic bulk tissue samples clustered separately from the cored samples (Fig. 2B).

Compared to the AT of the same sample type using unpaired Student’s *t*-test, bulk tumors had 1,668 differentially abundant proteins and tumor epithelial-enriched cores had 393 differentially abundant proteins. Proteins that were increased in both bulk tumor tissue and cored tumor epithelium were enriched in epithelium morphogenesis and ossification GO Biological processes, while proteins decreased in tumor compared to AT were enriched in Digestion and Vitamin metabolism (Fig. 2C). There were no significant differences in protein abundance in stroma-enriched cores from tumor and AT (Fig. 2D), nor were there significant differences between tumor stroma-enriched cores and tumor epithelial-enriched cores (Fig. 2E).

*KRAS* missense mutations were identified in the bulk and/or epithelial-enriched cored tumor samples in 14 patients. For 7 patients, the *KRAS* variant allele frequency (VAF) increased by at least 20% in the epithelial-enriched cores compared to bulk tissue (Fig. 2F). Additionally, the stromal-enriched cores of 7 patients had a minimum 20% lower *KRAS* VAF compared to the epithelial-enriched cores (Fig. 2F). Overall, BioTExt coring provided high quality multi-omics data but only half of the samples demonstrated an enrichment of a targeted cell type.

### Cell percentages vary at depth in tissue cores

**Fig. 3:**
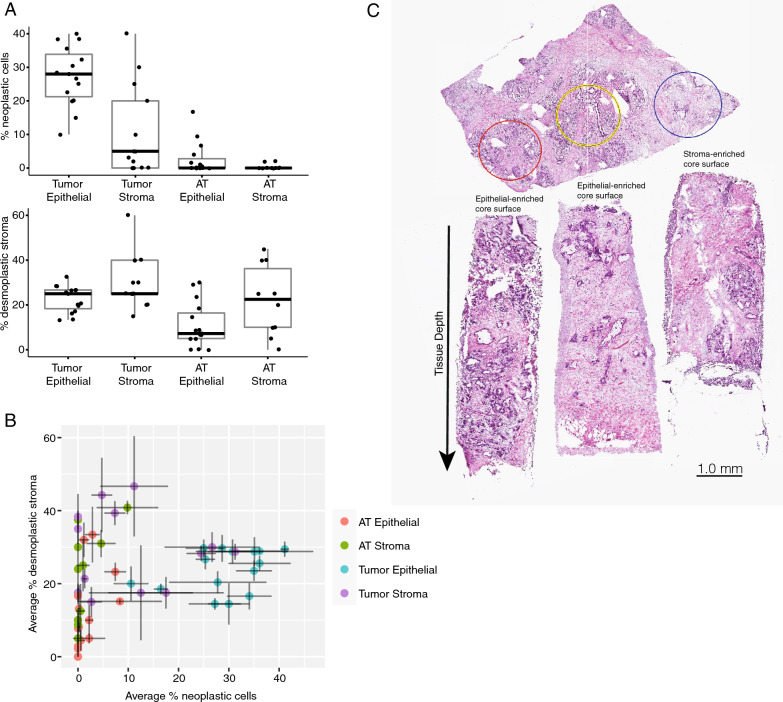
Tissue composition varies with core depth. **A** Percent neoplastic cells and desmoplastic stroma at the surface for each set of cores. **B** Average neoplastic cells percentage and desmoplastic stroma percent for each cored sample. Error bars are the standard deviation over at least three depths within the core. **C** (Top) OCT embedded PDAC frozen tissue cryosectioned at 5 µm and H&E stained with study pathologist selecting tumor rich (red, yellow) and stroma rich (blue) areas. (Bottom) Cryocores of selected regions 1.5 mm diameter with depths ranging from 4 to 7 mm placed en face in OCT

We hypothesized the tumor-enriched cores were not significantly different from the stroma-enriched cores due to our limited capability to physically extract pure neoplastic cell components. This limitation is a function of the exceedingly heterogeneous nature of PDAC tissue, where regions of pure epithelial or stromal tissue may comprise only a small fraction of the tissue and vary along the core depth. While regions selected for coring may contain one tissue type at the surface of the tissue block, deeper layers may not contain the same cellular constituents. For example, the tissue compartment targeted by the coring procedure may diminish further into the block. Although the percentage of neoplastic cells and desmoplastic stroma in the original slide assessed by H&E staining were significantly different between the tumor epithelial-enriched cores and the tumor stroma-enriched cores, there was significant overlap between the two with some stroma-enriched cores containing as much neoplastic epithelial tissue as the tumor epithelial-enriched cores (Fig. 3A). To quantify this variation, we calculated cell type percentages every 80 µm of core depth. We found that some cores containing tumor stroma in the top H&E had higher average percentages of neoplastic cells than some cores containing tumor epithelium at the surface, with cell type percentages that varied greatly across the tissue depth (Fig. 3B). For example, from one tumor block, the regions selected for coring had high neoplastic epithelium for the tumor-epithelial enriched cores and high stroma content for the stroma-enriched core according to the surface histology, but histology performed down the depth of the core showed variable regions of stroma and epithelium throughout the entire core (Fig. 3C). Therefore, classification of the samples as epithelial-enriched or stroma-enriched was confounded by tissue heterogeneity and could not be solely assigned based on the surface histology.

### Sample clustering using serial histology counts

**Fig. 4:**
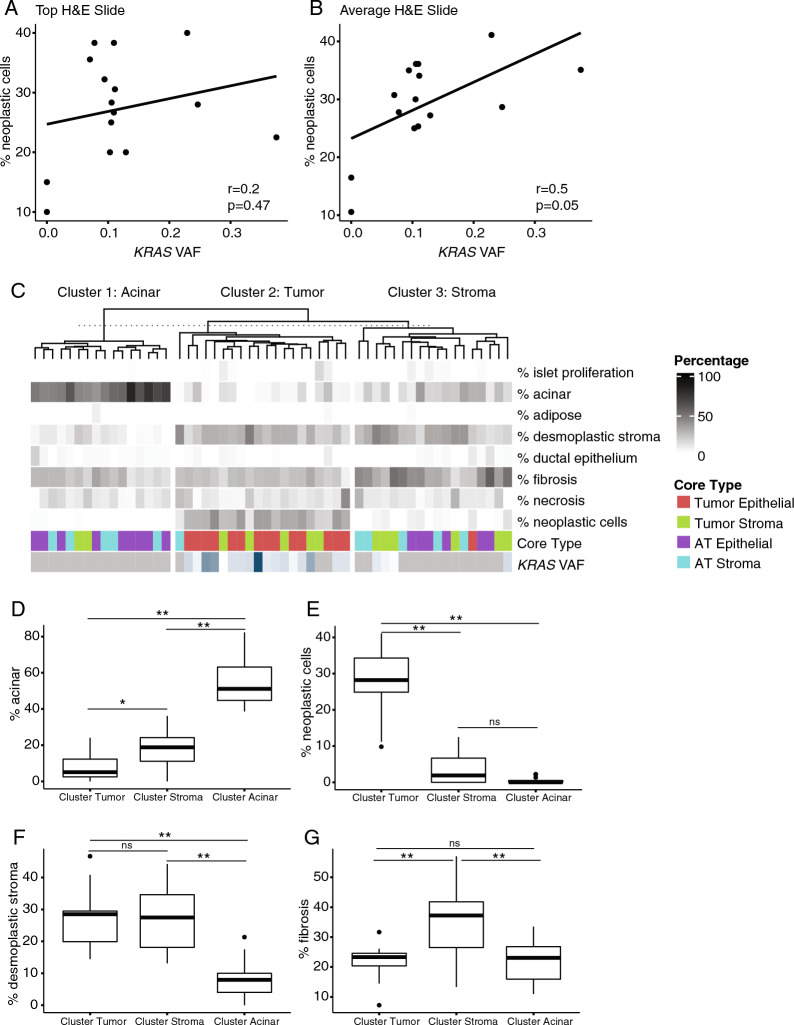
Sample clustering by average histology counts. **A** Percent neoplastic cells correlated with *KRAS* VAF for the surface H&E slide and **B** an average over 3 serial H&E slides. **C** Tumor clustering using percent cell types assessed by histology and averaged over multiple slides taken throughout the core. **D** Average percent acinar cells, **E** neoplastic cells, **F** desmoplastic stroma, and **G** fibrosis for the three histology clusters. *p < 0.005, **p < 0.0005

We observed no correlation between percent tumor content from the surface H&E slide and *KRAS* VAF in epithelial-enriched cores (Fig. 4A). However, the average percent tumor content from slides taken throughout the core had a strong positive correlation with *KRAS* VAF (Fig. 4B). In order to more accurately separate samples by tissue type, we used KNN clustering to group the cored samples into three groups using average histology percentages of cell types instead of the original assignment based on surface histology (Fig. 4C). The first cluster contained samples with a high percentage of acinar cells, which mostly included AT stroma-enriched cores and AT epithelial-enriched cores (Fig. 4D). The second group had the highest percent neoplastic cell content and consisted of the tumor cores of either type (Fig. 4E). The third group had the highest amount of fibrosis (Fig. 4F) and both the second and third groups had higher desmoplastic stroma than the acinar group (Fig. 4G). The three groups represented normal pancreatic tissue, enrichment of tumor content, and enrichment of stroma content.

### Identifying tumor epithelial-specific markers

**Fig. 5:**
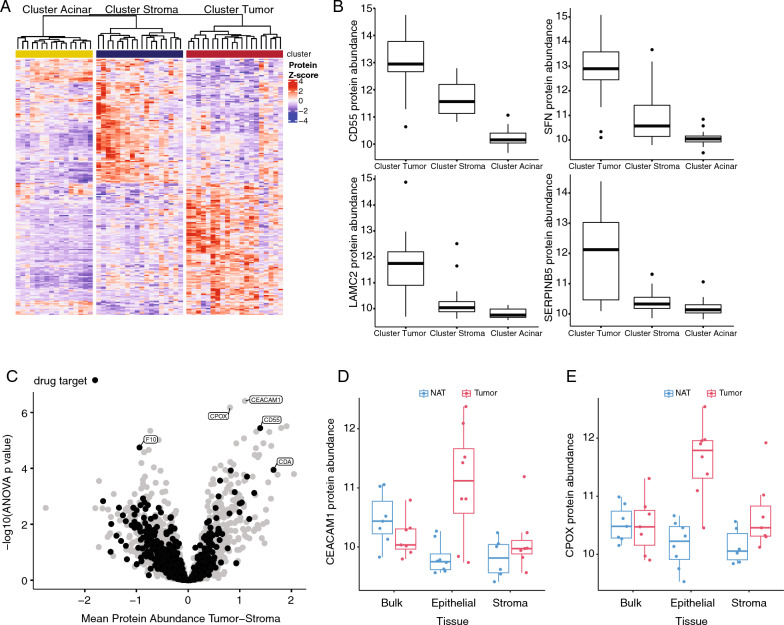
Proteomics differences in tumor vs stroma. **A** Proteomics abundance z score for proteins significantly different between the tumor and stroma clusters. **B** Protein abundance for four proteins that were identified as tumor-epithelial specific in the CPTAC PDAC study. **C** Protein abundance in tumor vs stroma clusters including proteins annotated as drug targets. **D** CEACAM1 protein abundance in the samples with a *KRAS* variant allele frequency increased > 20% in the epithelial-enriched core compared to the stroma-enriched core and bulk tissue. **E** CPOX protein abundance in the samples with a *KRAS* variant allele frequency increased > 20% in the epithelial-enriched core compared to the stroma-enriched core and bulk tissue

The primary histological differences between tumor and stroma clusters were tumor epithelial content and fibrosis. To identify putative tumor- and stroma-specific markers, we evaluated significantly different proteins across the clusters (ANOVA with Tukey’s post hoc comparison p value < 0.01 between the tumor epithelial group and the stroma group). Two hundred eighteen proteins were significantly different between the two groups (Fig. 5A). Of these, 110 (50%) were also upregulated in bulk tumors compared to AT in the earlier CPTAC PDAC study [5]. In the CPTAC PDAC study, 21 proteins were identified as tumor epithelial-specific using stringent criteria and 10 of these were significantly upregulated in the tumor cluster compared to the stroma cluster in this study. The top four were CD55, SFN, LAMC2, and SERPINB5 (Fig. 5B).

The cell of origin of overexpressed genes is an important consideration for therapeutic drug targeting. We downloaded the list of drug targets from Broad’s Drug Repurposing Hub and compared them to the BioTExt proteomics data. Seventeen genes increased in the tumor cluster and 23 genes increased in the stroma cluster were drug targets (Fig. 5C). These included CDA and CD55, which were upregulated in the tumor cluster and F10 upregulated in the stroma cluster. CDA can be targeted by cedazuridine and F10 can be targeted by several anticoagulants.

Additionally, CEACAM1 and CPOX were highly upregulated in the tumor cluster compared to the stroma cluster (Fig. 5C). To determine whether these proteins were upregulated specifically in tumor tissue, we selected samples where coring provided an enrichment of tumor epithelium or tumor stroma over the bulk tissue from the same patient. We defined enrichment as either a > 20% increase (tumor epithelium) or > 20% decrease (tumor stroma) in *KRAS* VAF compared to the bulk tumor tissue sample of the same patient. CEACAM1 was upregulated in cored tumor epithelial tissue (Fig. 5D) but the tumor/AT difference was not seen in bulk tissue. Similarly for CPOX, there was no difference in abundance between the AT and tumor bulk tissue, but there was a strong increase in CPOX abundance in cored tumor epithelial samples compared to cored AT (Fig. 5E). These results show that bulk tissue analysis may miss proteins specifically overexpressed in tumor epithelium.

## Discussion

The generation and integration of multi-omics data on a set of tissue samples has the potential to understand the consequences of genomic alterations and identify new avenues for treatment of disease [5]. However, one of the greatest challenges in studying PDAC is the heterogeneous composition of this type of tumor, combined with low tumor content of neoplastic cells. The high percentage of non-epithelium tissue in bulk tissue samples obscures signals in multi-omics data and it can be difficult to understand the contributions of the tumor microenvironment compared to neoplastic cells to the biology of the disease. Using the BioTExt coring technique, we separated bulk tissue into epithelial and stromal components to better understand the tumor microenvironment. Despite the limited amount of extracted tissue, we were able to generate high quality WGS, RNAseq, and DIA proteomics data with nearly 5000 proteins quantified. This demonstrates that comprehensive measurements can be acquired even on small tissue samples.

However, the tissue cores reflected the issue of mixed cell populations seen in bulk tissue as tissue composition rapidly differed below what was seen at the surface. To account for this issue, we used serial histology sectioning to assess cell type percentages throughout the depth of the core. The average histology cell type percentages demonstrated a better correlation with *KRAS* variant allele frequency, showing that the small area used as selection from the surface slide did not reflect the biology of the entire core. Histology subtyping allowed us to better separate the cores into samples with enrichment of neoplastic epithelium, stroma, and acinar cells and identify tumor- and stroma-specific markers.

Using the histology subtyping with BioTExt coring, we identified an increase in abundance of two proteins, CEACAM1 and CPOX, in the neoplastic epithelium that were not identified in the bulk tissue analysis. CEACAM1 is a cell adhesion molecule that is present in epithelial cells throughout the body [21] and has been shown to localize to neoplastic epithelial cells in pancreatic adenocarcinoma [22]. *CPOX* encodes the enzyme coproporphyrinogen oxidase, which is involved in the biosynthesis of heme and has not been studied much in the context of cancer. However, heme synthesis is important in PDAC cell proliferation [23] and serves as a metabolic vulnerability in PDAC in vivo [24]. Because CPOX is primarily involved in metabolism, it may be interesting to further explore bioenergetic differences between epithelium and stroma. Together they highlight proteins that may contribute to tumorigenesis in PDAC but that may be obscured in bulk tissue analysis. Future work could confirm the role of CEACAM1 and CPOX in PDAC and immunohistochemistry could be used to support the epithelial stratification.

While this study highlights the utility of the BioTExt coring method in combination with serial histology to generate and harness multi-omics data specifically in PDAC, this same method could be useful in many other cases. The BioTExt coring technique was pioneered for use on small tissue samples, such as core biopsies from breast cancer [15]. However, it could also be used for other highly heterogeneous tumor types, such as prostate cancer. Finally, BioTExt coring may help separate the epithelial component in cases of other tissues where the percentage of epithelium is small compared to other tissue types. For example, normal adjacent tissue in colon and endometrium primarily consist of muscle tissue with a small fraction of epithelial tissue and separating out the normal epithelium will facilitate the comparison with neoplastic epithelium. There are some shortcomings to using the method outlined here. The coring with the serial histology is still time consuming, requires the help of expert pathologists, and does not extract pure tissue components. These challenges could be mitigated in the future by improved algorithms and methods. Better extraction might occur with initial macrodissection. Computer algorithms or other imaging techniques may also be developed to select regions for coring and automatically quantify different cell types in histology images. Automated histology analysis is already being developed to distinguish tumor from stroma [25] and with the continued advancements in deep learning algorithms this option should only improve in the future.

## Conclusions

BioTExt coring in combination with serial histology facilitates cell type-aware proteogenomic characterization of PDAC. The coring technique provides enough material for high quality multi-omics data generation that can be used to develop new hypotheses for signaling involved in PDAC progression and opportunities for treatment.

### Supplementary Information

**Additional file 1:** **Table S1.** Aliquot IDs for the BioTExt samples.

**Additional file 2:** **Table S2.** RNA sequencing, proteomics, histology, and KRAS mutation data.

## Data Availability

The genomics and transcriptomics datasets generated in this study are available at the Genomics Data Commons (https://gdc.cancer.gov/) and the proteomics datasets are available at the Proteomics Data Commons (https://pdc.cancer.gov/, PDC000504). Processed data are available as supplementary files in this article.

## Abbreviations

AT: Pancreatic tissue collected from a region adjacent to neoplastic tissue
BioTExt: Biopsy Trifecta Extraction Technique
BCR: Biospecimen Core Resource
CPTAC: Clinical Proteomics Tumor Analysis Consortium
DIA: Data-independent acquisition
FFPE: Formalin-fixed paraffin-embedded
GDC: Genomics Data Commons
KNN: K-nearest neighbors
OCT: Optimal cutting temperature compound
PDAC: Pancreatic ductal adenocarcinoma
TSS: Tissue Source Site
VAF: Variant allele frequency
WGS: Whole genome sequencing
